# Improving Theatre Productivity by Digitising Surgical Equipment Repairs

**DOI:** 10.7759/cureus.61802

**Published:** 2024-06-06

**Authors:** Pranai Buddhdev, Jenny Tebby, Peter Black, Davina Harding, Janet Kendall, Heer Shah

**Affiliations:** 1 Orthopaedics and Trauma, Mid and South Essex NHS Foundation Trust, Essex, GBR; 2 Sterile Services, Mid and South Essex NHS Foundation Trust, Essex, GBR; 3 Emergency Medicine, St George's University Hospitals NHS Foundation Trust, London, GBR

**Keywords:** equipment maintainance, sterilization, hospital management, theatre efficiency, theatre productivity, instrument repairs, digital health, surgical instrument

## Abstract

Introduction

A few cancelled surgeries are due to surgical equipment issues representing a significant burden to both patients and National Health Service (NHS) hospitals on waiting lists. Despite this, there remain very few strategies designed to tackle these avoidable cancellations, especially in combination with digitisation. Our aim was to demonstrate improved efficiency through a pilot study in collaboration with Broomfield Hospital (Broomfield, United Kingdom), MediShout Ltd (London, United Kingdom), and B. Braun Medical Ltd (Sheffield, United Kingdom) with the digitalisation of the equipment repair pathway.

Methods

MediShout digitised two distinct repair pathways: ad-hoc repairs and maintenance equipment services (MES). Pre- and post-digitisation outcome measures were collected including the number of process steps, staff contribution time, non-staff continuation time, turnaround time, cancelled surgeries, planned preventative maintenance compliance, and staff satisfaction. The number of steps, staff contribution time, and non-staff contribution time were calculated using cognitive task analyses and time-motion studies, respectively. Turnaround time and cancellation data were taken from existing hospital data sets and staff satisfaction was measured through two staff surveys.

Results

Digitising the ad-hoc repair pathway reduced the number of steps by 18 (118 to 100) and saved 74 minutes of total staff time (Broomfield Hospital and B. Braun) per repair, resulting in annual efficiency savings of £21,721.48. Digitising the MES repair pathway reduced the number of steps by 13 (74 to 61) and saved 56 minutes of total staff time per repair, resulting in annual efficiency savings of £3469.44. Turnaround time for the repaired kit decreased by 14 days and 29 days for the digital ad-hoc and digital MES pathways, respectively. Elective operations cancelled due to equipment issues decreased by 44%, from 1.5 operations/month pre-pilot to 0.83 operations/month post-pilot. Planned preventative maintenance compliance across the MES pathway increased by 67% (33% to 100%). Staff satisfaction with the repair pathway improved from 12% to 96%.

Conclusion

This pilot study showcases the numerous benefits that can be achieved through digitisation and offers an innovative case study to approach avoidable cancellations due to equipment failure.

## Introduction

Cancellation of surgical cases due to numerous patient, hospital, and logistical factors is common and represents an unnecessary cost to patients awaiting care, hospitals delivering this care, and the system at large. The psychosocial impact of cancellations has been well documented previously, with patients commonly expressing increased feelings of stress, anxiety, and anger [1-3]. Equally, cancellation of elective operations prolongs a patient’s waiting time, correlating with increased pain and use of over-the-counter (OTC) analgesics, poorer quality of life scores, and increased postoperative complications [3-5]. Several studies have highlighted the financial costs of cancellations for patients. These include loss of employment, additional travel costs, and costs associated with organising extra childcare [3-6].

The incidence of cancelled operations across the United Kingdom (UK) ranges from 5% to 14% but has been reported closer to 40% in other parts of the world [7-10]. This reflects a poor use of hospital resources from under-utilised operating rooms and staff. Moreover, despite the lack of data available, the loss of income from cancellations is estimated to be between £1-3.2 million per NHS hospital or around £400 million nationally per year [11,12]. An NHS Institute of Innovation and Improvement report approximated the cost of a four-hour operating session to be £2818.96, accounting for theatre staff, estates, and equipment. NHS hospitals fund all their available operating sessions but underutilisation results in approximately £2.2 million of additional expenditure [13]

There are a number of reasons for cancelled operations. Causes of unavoidable cancellations include patient refusal or concerns over their anaesthetic safety due to medical illness or co-morbidities. However, a significant proportion of cancellations stem from preventable causes such as administrative errors, bed unavailability, poor scheduling, or equipment issues. Three studies investigating reasons for cancelled operations across UK hospitals found the percentage of cancellations attributed to preventable causes was often more than 50% [7-9].

There is a growing urgency for hospitals to adopt strategies that both minimise and prevent cancellations. The introduction of preoperative clinics and telephone text message reminders have helped patients familiarise themselves with their upcoming surgeries, reducing non-attendances [14-16]. Similarly, allocating accurate times for surgeries based on patient characteristics such as age and complexity has been shown to reduce the over-running of operating lists [17]. More recently, Sherwood Forest Hospital in Mansfield, UK, incorporated the telehealth solution ‘Florence’ (Generated Health Ltd, London, UK) into their preoperative pathway; this tool helped identify hypertensive patients ahead of their surgeries, allowing proactive management measures to be taken [18]. While such solutions are undoubtedly beneficial, there remains a clear lack of innovation towards other causes of cancellations such as instrument failure, especially in combination with digital technologies.

Broken or missing surgical instruments are responsible for up to 4% of unnecessary cancellations each year [7-9]. Instruments are not only subjected to daily wear and tear during operations but also suffer damage due to misuse and careless transportation. Moreover, sterilisation leaves instruments vulnerable to heat/cool cycles and erosive cleaning processes that accelerate degradation [9]. Despite this, reports from the NHS and the Getting It Right First Time (GIRFT) initiative show that repairs which are conducted on an ad-hoc basis, where each equipment fault triggers a multi-step process, including procurement tasks, continue to be the current standard [19-21]. The high administrative burden caused by these workstreams causes delays to equipment availability and subsequently increases cancelled operations [22].

In contrast, managed equipment service (MES) contracts provide a service for surgical instruments for a fixed annual cost. Faulty instruments are sent for repair promptly and the predictable nature of service requests alongside a simplified procurement process can lead to improved workflow and financial planning, plus a swifter return of repaired instruments [20]. Additionally, instruments can be sent for preventative maintenance servicing after a specific timeframe or pre-determined number of uses as per the manufacturers’ recommendation, improving their longevity. Although MES contracts are not a new concept, adoption within the NHS has been slow, with reluctance, often centering around the premium cost of MES contracts despite some studies indicating they can contribute a 5-10% cost-saving across an instrument’s lifespan [21].

There are very few studies investigating the operational and financial benefits of MES contracts when compared to ad-hoc requests. In this study, we aim to better understand the processes involved with these pathways, whilst analysing the potential benefits of digitising these pathways.

## Materials and methods

Clinical setting

This prospective study was conducted over six months, starting from August 2023, at Broomfield Hospital (Mid & South Essex NHS Trust), Broomfield, UK. Damaged surgical instruments were processed by the Sterile Services Department (SSD) and repaired by B. Braun Medical Ltd (B. Braun, Sheffield, UK), with whom the hospital has repair contracts for sterile goods management. Most instruments were repaired on an ad-hoc basis once they had been reported as damaged. A smaller proportion of instruments were under an MES contract and were repaired periodically within set time windows.

Over the course of the pilot, traditional reporting pathways for both ad-hoc and MES repairs were replaced by digital reporting, where hospital and B. Braun staff used a digital platform called MediShout (MediShout Ltd, London, UK) to communicate. MediShout is a digital platform which streamlines operational pathways within hospitals. 

Traditional repairs process: process mapping and time-motion study (TMS)

Each step of the traditional repair process for both ad-hoc and MES repairs was noted down and mapped. Any deviations, defined as steps that occurred frequently but not always, were also included. Each deviation was assigned a probability to determine the likelihood of occurrence. Multiple staff members verified each deviation's sequential steps, deviations, and probability. 

A TMS measured staff contribution and non-staff contribution time on each task. Two observers (HS and CM) used a stopwatch to calculate the time to complete a task on three occurrences. The average time taken for each step was calculated and documented. Non-staff contribution was also measured for time elapsed between stages of the repair process. In cases where the time elapsed varied, staff members were interviewed, and averages were taken. 

MediShout’s digital software: process mapping and TMS

MediShout’s mobile application and software replaced the traditional repair processes. Each staff member was assigned a unique username and password to access the digital platform. Staff members could request new repairs and track the progress of each repair through its repair cycle. Additionally, paper and PDF (portable document format) forms such as the ‘decontamination certificate’ and ‘repair quote’ were embedded into each repair digitally such that all key information was available in one place. As the instrument progressed through each stage of the repair cycle, a new status and accompanying notification were triggered on MediShout. Users had the ability to adjust notification settings in order to receive those pertinent to their job role. An embedded chat feature allowed users to communicate queries or raise issues directly through the digital platform. Moreover, users could export completed repairs on a weekly, monthly, or annual basis directly from the platform. Process mapping and TMS were subsequently repeated with MediShout’s digitised repair pathways to compare them to the traditional repair processes.

Efficiency savings

Each staff member was assigned a cost-per-hour value to help estimate the cost of the traditional and digital repair processes. Cost-per-hour estimates for B. Braun staff were provided by B. Braun Medical Ltd members and for NHS staff by NHS managers. Efficiency savings were calculated by subtracting staff contribution times for traditional and digital repair processes, multiplied by their hourly rate and average annual repair volume. To compare these benefits, the average yearly volume for both ad-hoc and MES contracts was extrapolated from paper records spanning the previous year. For ad-hoc repairs, we estimated an average volume of 12 per week and for MES repairs we estimated an average volume of four per week. 

Turnaround time

Turnaround time was defined as the number of days elapsed between a processed repair and the return of the repaired instrument into circulation. For traditional ad-hoc and MES repairs, the turnaround time was calculated using archived paper records from the SSD of all processed repairs between January 1, 2023, and August 31, 2023. For repairs following the introduction of MediShout’s software, turnaround time was computed using exported reports with timestamps for the first and final steps of the repair process. Turnaround time was calculated between October 14, 2023, and January 31, 2024.

Planned preventative maintenance

For digital MES repairs, MediShout integrated with Health Edge Solid-State Drive Application (HESSDA) (Health Edge Solutions Ltd, Bristol, UK) application programming interface (API), which tracked the number of uses of each asset. Assets nearing a set number of uses (n=40) would trigger a notification on MediShout indicating the need for a service. This introduced a repair service based on use, replacing the existing process which serviced MES instruments based on a set timeframe (12-monthly).

Measuring staff satisfaction

Staff surveys were conducted before and after MediShout’s digitisation (See Appendices). The surveys were conducted using the MediShout App and delivered to staff members within the SSD and B. Braun’s Customer Support team. Additional information including the respondent’s role and name was also collected.

Pre-Implementation Survey

Staff members were asked three questions to understand their views, opinions, and any frustrations with the traditional repair process. Each question also had an optional white space for respondents to provide additional information and context. 

Post-Implementation Survey

Staff members were asked four questions on their opinions following MediShout’s digitisation of both the ad-hoc and MES repair pathways as well as any future improvements that would be valuable. 

The second survey was conducted after the software was launched. It helped to understand any new challenges as well as any improvements made with regard to time-saving, ease of use, and overall satisfaction. 

Statistical analysis

A two-tailed T test was conducted to compare both the traditional and MediShout processes for staff contribution time and turnaround time across both ad-hoc repairs and MES repairs. A significance level of 0.05 was used for all analyses. 

## Results

Process mapping and TMS

Traditional Versus Digital Ad-Hoc Repairs

The traditional repair process comprised 101 steps and 17 possible deviations. These were divided into eight separate stages spanning the hospital (Broomfield Hospital) and service company (B. Braun). The cumulative recorded time to complete a single repair was 734 hours and 19 minutes (30 days).

**Figure 1:**
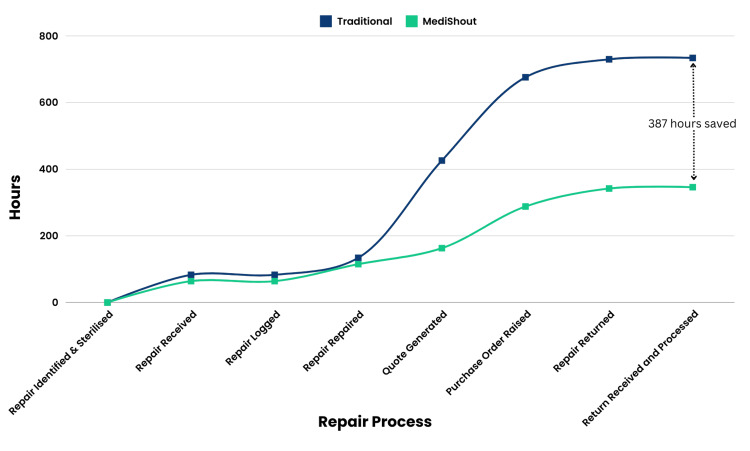
Time-motion study of traditional versus digital ad-hoc repairs (cumulative hours)

The digitised reporting process for ad-hoc repairs comprised 95 steps and six possible deviations, equating to a total step reduction of 18%. Recorded time decreased by 53.4%, totalling 346 hours and 26 minutes (14 days), a saving of 16 days. Moreover, time was saved across seven of the eight areas, with the most significant time savings seen in ‘Repairs Received’ and ‘Quote Generated’ due to substantial reductions in time-intensive deviations (Figure 1). 

Traditional Versus Digital MES Repairs 

The traditional process for MES repairs consisted of 63 steps and 11 deviations. These were divided into seven separate stages spanning Broomfield Hospital and B. Braun. The cumulative recorded time to complete a single repair was 243 hours and 34 minutes (9 days). 

**Figure 2:**
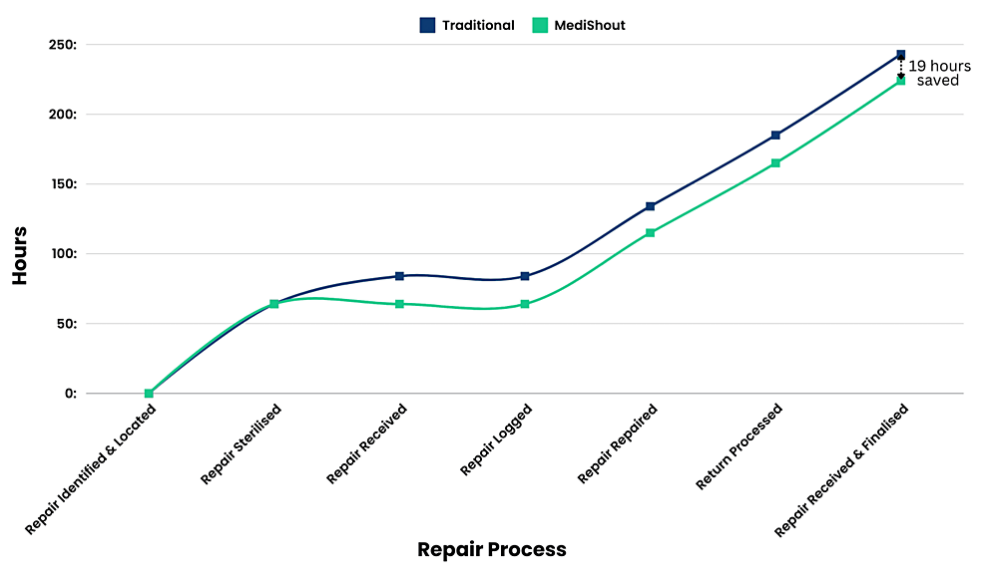
Time-motion study of traditional versus digital MES repairs (cumulative hours) MES: maintenance equipment services

The digitised MES repair process consisted of 55 steps and six deviations, a step reduction of 18%. Recorded time decreased by 8% to 224 hours and three minutes (nine days), a saving of 19 hours. Implementing MediShout’s solution saved time across four areas, with the most significant time saving seen in ‘Repairs Received’ due to the reduction in deviations. Another considerable time saving was seen during the ‘Repair Logged’ process due to digitisation and subsequent removal of existing B. Braun ‘TS sheets’ (Figure 2). A summary comparing both repair processes is shown in Table 1. 

**Table 1:** Time motion study comparison between digitised and traditional repair processes MES: maintenance equipment services

Repair process	Number of steps	Number of deviations	Cumulative hours
Ad-Hoc	101	17	734.3
Digital Ad-Hoc	94	6	346.5
MES	63	11	243.5
Digital MES	55	6	224

Staff time contributions and non-cash-releasing benefits

Traditional Versus Digital Ad-Hoc Repairs

The ad-hoc repair pathway involved seven distinct personas, each concerned with a separate stage of the repair. Each traditional ad-hoc repair took an average of two hours, 10 minutes, and 58 seconds of staff time, resulting in a total labour cost of £78.05. In contrast, each digital ad-hoc repair took an average of 56 minutes and 47 seconds, resulting in a total labour cost of £43.24 and a cost saving of 45%. 

On average, Broomfield Hospital sends 12 instruments for repair to B. Braun each week. Extrapolating this out across a 52-week year, adopting a digital pathway would result in a staff time saving of 771.5 hours a year (32 days), equating to potential efficiency savings of £21,721.48. The most significant savings were for SSD technicians (SSD staff) and staff within the Customer Support Team at B. Braun. 

Traditional Versus Digital MES Repairs

The MES repair pathway bypassed the Procurement and Finance teams due to contractual agreement. Each MES repair took one hour, 50 minutes, and 22 seconds on average, resulting in a total labour cost of £58.02. In contrast, each digital repair took 53 minutes and 46 seconds on average, resulting in a total labour cost of £41.34 and a cost saving of 29%. 

On average, Broomfield Hospital sends four MES instruments to B. Braun each week. Extrapolating this out across a 52-week year, adopting a digital MES pathway could save potentially 196.2 staff hours annually, equating to potential efficiency savings of £3469.44. Reduced staff time and cash-releasing savings were seen across all staff members except the engineering team, whose repairs process was unaltered by the MediShout solution. A summary is shown in Table 2. The results show a statistically significant difference between MediShout's digital pathways and the traditional pathways (p<0.05). 

**Table 2:** Staff contribution and cost comparisons between digitised and traditional repair processes *Extrapolated at current repair volumes MES: maintenance equipment services

Repair process	Staff contribution time for one Item (hour:minute:second)	Staff cost for one Item (£)	Estimated annual staff cost (£0*	p-value
Ad-Hoc	2:10:58	78.05	48 703.20	0.0215
Digital Ad-Hoc	0:56:47	43.24	26 981.76	
MES	1:50:22	58.02	12 068.16	0.0475
Digital MES	0:53:46	41.34	8598.72	

Turnaround time

Ad Hoc Repairs: Traditional Versus Digital Pathways

**Figure 3:**
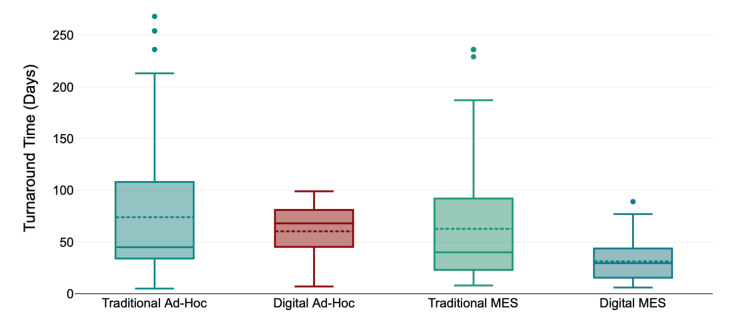
Turnaround time of traditional versus digital repair pathways MES: maintenance equipment services

In total, 127 repairs were sent to B. Braun between January 1, 2023, and August 31, 2023. Repairs were sent from eight departments, with General Surgery contributing the highest volume of repairs. Turnaround time ranged from 15 to 268 days (mean 74, median 45). Via the digital reporting solution, 118 instrument repairs were processed between August 29, 2023, and January 24, 2024. Turnaround time ranged from seven to 99 days (mean 60, median 68), thus a reduction of 19% or 14 days (Figure 3). 

MES Repairs: Traditional Versus Digital Pathways

A total of 108 MES repairs were sent to B. Braun Medical Ltd between January 1, 2023, and August 31, 2023, using the traditional process. Turnaround time ranged from eight to 236 days (mean 63, median 40). Via the digital reporting solution, 55 MES repairs were processed between November 6, 2023, and February 8, 2024. Turnaround time ranged from six to 89 days (mean 31, median 29), thus a reduction of 48% or 28 days (Figure 3). 

Digital Repair Pathways in 2024

As users gain familiarity with the digital repair pathway, turnaround time will continue to improve; in the first two months of 2024, 12 ad-hoc and eight MES repairs were processed. The average turnaround times were 39 days and 16 days, respectively, representing a 47% and 73% reduction from the respective traditional pathways (Table 3). While digitisation reduced turnaround time across both pathways, these were not statistically significant (p>0.05). 

**Table 3:** Turnaround time of traditional versus digital repairs MES: maintenance equipment services

Repair process	Number of repairs	Average turnaround time (Days)	p-value
Ad-Hoc	127	74	0.1697
Digital Ad-Hoc	118	60	
MES	108	60	0.3229
Digital MES	55	31	

Reduction in cancelled surgeries

Between January 1, 2023, and August 31, 2023, 12 operations were cancelled on the day due to equipment issues (1.5/month). Following MediShout’s involvement with the repair pathways, five equipment-related cancellations were recorded between September 1, 2023, and February 29, 2024 (0.83/month), a 44% reduction of monthly cancellations due to equipment issues. 

Planned preventative maintenance

An analysis of 167 sets under MES contracts found that 67% were not serviced within their recommended timeframe. Eight sets were last serviced over 100 months ago, and one set hadn’t been serviced in close to 11 years. The new digital MES pathway prompts service requests for sets based on the use volume. MediShout notifies staff members when sets approach 40 uses, prompting a service repair. Since digitising, all sets that required service have been processed within their recommended use limit (100% compliance).

Staff satisfaction

A total of 24 staff members were surveyed In August 2023, on their thoughts and experiences with the traditional repair pathways. Of these, 33% of respondents were dissatisfied, 55% were neither dissatisfied nor satisfied with the existing processes, 71% said that the authorisation process was the most difficult, and 92% of respondents said that digital repair pathways would be useful (29%) or very useful (63%) to them. 

**Figure 4:**
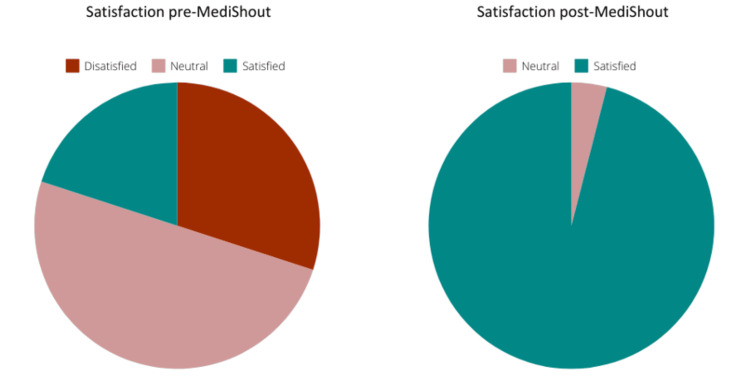
Satisfaction prior to and post MediShout

A second survey following the launch of digital repair pathways was conducted in October 2023. Ninety-six per cent of respondents were either very satisfied (71%) or somewhat satisfied (25%) with the digital repair pathways, 88% of Respondents felt the digital solution had improved the authorisation process, and all respondents thought that the digital solution had made the progress of each repair easier to track. Similarly, all respondents stated they would recommend the digital solution to other business units or repairs (Figure 4). 

## Discussion

Digital repair pathways

Digitising the theatre equipment repair processes has delivered several significant benefits; across both pathways, digitisation has been shown to reduce the number of steps, staff contribution time, and frequency of deviations. Fewer steps created fewer points of failure across the entire process, improving efficiency. Moreover, reduced contribution time boosted productivity as staff members could use the saved time to tackle other pertinent tasks. Similarly, the reduced contribution time highlights the considerable non-cash-releasing benefits of £25190.88 after implementing digital repair pathways.

Increased planned preventative maintenance compliance

MES contracts aim to keep all instruments functioning optimally by providing a recurring service aligned to the manufacturer’s gold standard. However, maintenance services were often delayed or missed altogether due to the difficulty tracking usage and constant demand for kits. This further increased the likelihood of suboptimal intra-operative performance and future equipment failures. Digitising the service pathway automatically tracked usage, replacing the existing paper tracker and providing early warnings to managers of kits approaching their service threshold. These allowed for staff to better plan future kit allocations for upcoming surgeries. Since digitisation, compliance with planned maintenance dates increased from 33% to 100%, highlighting the benefits of a digital MES pathway. Expanding the digitised planned preventative maintenance pathway across all repairs would increase the lifespan of surgical kits while also reducing the placement rate creating further non-cash-releasing benefits. 

Improved theatre efficiency

Digitising the repair pathways significantly reduced turnaround time to 60 days for the digital ad-hoc pathway and 31 days for the digital MES pathway, on average. Therefore, switching to an entirely digital MES pathway would therefore give hospitals a greater supply of available kits for surgeries for more operating days each year. This in turn reduces pressure on the SSD, improves theatre efficiency and lessens the likelihood of cancellations due to kit shortages. Moreover, as staff members become more familiar with the digital platform and further updates are introduced, turnaround time will continue to decrease. 

Reduction in cancellations and cash-releasing savings

Cancelled surgeries are subsequently rescheduled within the next 28 days representing a significant logistical burden to booking managers. Moreover, rescheduled surgeries require the same staff and equipment resources representing a double cost to the hospital. Digitising repair pathways reduces the likelihood of cancellations due to equipment shortages creating cash-releasing savings for the hospital. 

The current pilot focused on four of the eight business units between B. Braun and Broomfield Hospital. Therefore, an extension of this pilot to these wider areas could bring further non-cash-releasing and cash-releasing savings. 

Wider benefits of a digital platform

Improved Staff Satisfaction and a Paperless Service 

Digitising the repair pathways streamlined communication channels, eradicated repetitive tasks and improved traceability. Consequently, staff satisfaction improved considerably, with all users stating their wish to see the digital platform rolled out to wider services. 

Additionally, each paper form was replaced with a digital counterpart approved by end users. This led to a richer flow of information from one party to the next. Moreover, the paperless pathways reduced the hospital’s carbon footprint creating social value benefits.

Improved Compliance and Safety

User-centric digital platforms are optimised to serve end-users and offer a range of more comprehensive benefits. Historically, 2% of instruments sent for repair did not have a matching decontamination certificate, voiding the repair agreement. Instead, an extra layer of security was added by ensuring matching decontamination certificates were a submission requirement for digitally processed repairs, thus improving compliance. Similarly, the traditional paper forms were quickly lost, often illegible and sometimes incomplete. The digital counterparts offer required fields, ensuring all necessary information is passed to the following user. Equally, all historic repairs are stored securely on a hosted server and can be downloaded for audit purposes at the user's convenience.

Limitations

We recognise several limitations with the current study. Firstly, the number of repairs processed following digitisation was lower than those prior to digitisation. This limits the reliability of any comparisons made or conclusions drawn. Secondly, there was significant variation in the reported turnaround time following digitisation. We hypothesised that as users gained familiarity with the new process, they were able to process and complete repairs faster. A more comprehensive post-digitisation analysis would be needed to confirm and validate this hypothesis. Lastly, variability in hospital repair processes across the NHS may limit the scalability of such solutions. 

## Conclusions

The collaboration between Broomfield Hospital, B. Braun, and MediShout represents an innovative approach to improving the current repair pathways. Our results highlight the significant benefits that can be achieved through digitisation within a short period. Although many digital implementation projects incur friction as users adopt the new software, the longer-term benefits vastly outweigh these short-term frustrations. Moreover, there is a growing need to introduce innovative projects that save costs, increase efficiency, and improve value across the entire NHS landscape. Therefore, we hope that this pilot study can be the springboard that fosters innovation across broader healthcare networks.

## Figures and Tables

**Figure 5:**
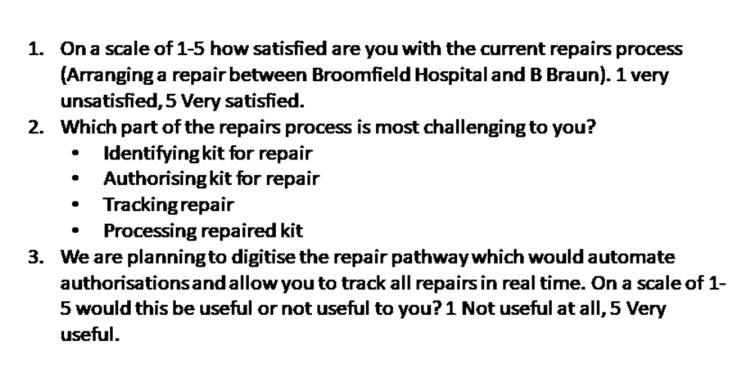
Staff survey before digitisation

**Figure 6:**
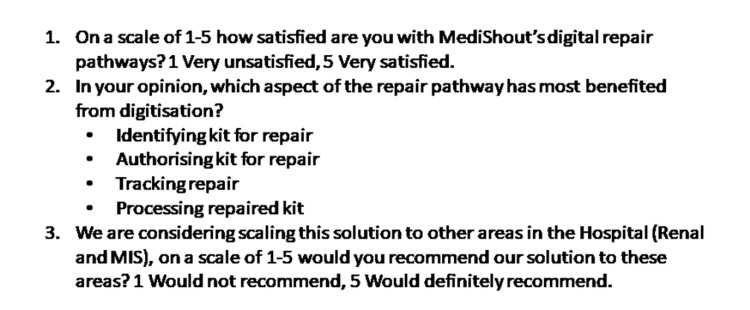
Staff survey after digitisation
